# Neural activity in human ventromedial prefrontal cortex reflecting the intention to save reward

**DOI:** 10.1093/scan/nsaa013

**Published:** 2020-01-28

**Authors:** Leopold Zangemeister, Fabian Grabenhorst, Wolfram Schultz

**Affiliations:** Department of Physiology, Development and Neuroscience, University of Cambridge, Downing Street, Cambridge CB2 3DY, U.K

**Keywords:** planning, choice, sequence, BDM, functional MRI

## Abstract

Saving behavior usually requires individuals to perform several consecutive choices before collecting the final reward. The overt behavior is preceded by an intention to perform an appropriate choice sequence. We studied saving sequences for which each participant rated the intention numerically as willingness to save. Each sequence resulted in a specific reward amount and thus had a particular value for the participant, which we assessed with a Becker-DeGroot-Marschak auction-like mechanism. Using functional MRI, we found that blood-oxygen-level-dependent signals in human ventromedial prefrontal cortex (vmPFC) correlated with the participant’s stated intention before each choice sequence. An adjacent vmPFC region showed graded activation that reflected the value of the sequence. These results demonstrate an involvement of vmPFC in intentional processes preceding sequential economic choices.

## Introduction

Planned, voluntary behavior requires the formation of explicit intentions (Lau *et al.,* 2004). The intention to forego an immediate reward in order to attain more reward in the future lies at the core of many behaviors, including economic saving (Benhabib and Bisin, 2005; Brown *et al.,* 2009). Humans typically devalue (or ‘temporally discount’) future rewards and often prefer smaller immediate to larger delayed rewards (Berns *et al.,* 2007). Furthermore, reward-directed intentions are often distinct from executed behavior as they can be overruled by decisions at a future time point leading to ‘time inconsistent’ decisions (Loewenstein and Prelec, 1992). Intentions for motor actions have been associated with parietal-frontal circuits (Sakai *et al.,* 2002; Andersen and Cui, 2009). Imagining future states through ‘episodic future thinking’ (also called ‘prospective’ thinking) has been associated with activation in ventromedial prefrontal cortex for a long time (cf. Schacter *et al.,* 2007).

A largely separate body of evidence implicates vmPFC in the subjective valuation of choice options. Blood-oxygen-level-dependent (BOLD) signals in vmPFC code the value of goods, choices and actions (Boorman *et al.,* 2009; Wunderlich *et al.,* 2010; Grabenhorst and Rolls, 2011; Rushworth *et al.,* 2011; Clithero and Rangel, 2014), temporally discounted subjective value (Kable and Glimcher, 2007) and relative attended value (Lim *et al.,* 2011). BOLD signals in this cortical structure can predict the degree to which thinking into the future modifies temporal discounting (Peters and Buchel, 2010), which provides a link to prospective aspects of value coding in vmPFC. However, little is known about the role of vmPFC in coding explicit intention and value during sequential reward-directed choices.

The current experiment employs the design of our previous study that dissociated planning from execution of choices using the same sequential task (Zangemeister *et al.,* 2016). The study had reported neural correlates for observable saving strategies in a major reward structure of the brain, the amygdala. However, despite additional activations in lateral PFC, we failed to find a specific involvement of the vmPFC in the rewarded task, despite its undisputed role in reward processing. To address the issue, we considered its role in intentions underlying rewarded behavior cited above. We had found some indications for such activities in the dorsolateral prefrontal cortex (dlPFC) and therefore carried out additional, more specific analyses to search for the coding of intentional processes in the vmPFC that could be related to sequential choices. In each step of a choice sequence, the participant chose between saving a liquid reward with interest and spending (i.e. consuming) the accumulated amount. Each sequence varied in length (number of saving steps to reward, chosen by the participant), type of reward (high-fat vs. low-fat milkshake, preset by the experimenter), and interest rate (high vs. low, preset by the experimenter). Before each sequence, participants stated the degree of their intention to engage in that sequence by numerically rating their willingness-to-save (WTS). Thus, the numeric WTS indicated the intention of each participant to save reward by engaging in a specific number of steps. To relate the intention to the valuation of each choice sequence *prior to performing it*, we asked participants to state their WTP for each sequence, using a Becker-DeGroot-Marshak (BDM) auction-like mechanism (Becker *et al.,* 1964). The ‘incentive compatible’ nature of BDM encourages truthful subjective valuation. Thus, the WTP, as estimated by the numeric BDM bid, indicated the subjective value of a specific sequence length for each participant. To evoke temptations and maximize reward tangibility, we used liquid rewards that participants consumed in the fMRI scanner immediately at the end of each sequence (McClure *et al.,* 2007; Brown *et al.,* 2009; Grabenhorst *et al.,* 2010b). We found, in advance of the sequential saving choices, distinct BOLD signals in vmPFC that reflected the participants’ intentions for performing the choice sequence, together with the corresponding sequence valuation.

**Fig. 1:**
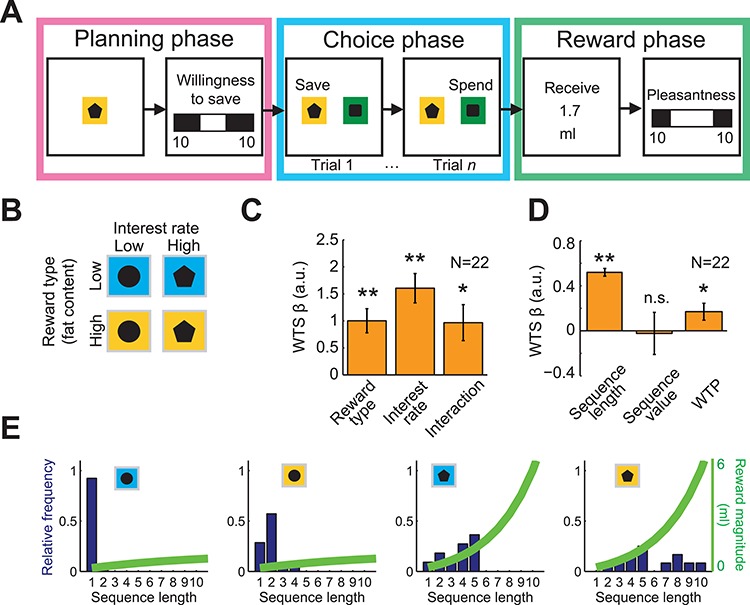
Economic-saving task and BDM auction-like mechanism for saving options. **A-B**, Economic-saving task. Participants performed choice sequences of self-defined lengths to save liquid rewards with different fat content that accumulated according to a given interest rate. In the planning phase, pre-trained cues indicated current interest rate and reward type, which varied sequence by sequence. Subsequently, they indicated their intentions to save in the forthcoming sequence (WTS) on a scale of 0–10 on a visual analogue scale (which increased simultaneously in both directions starting from the center to avoid a bias in visual left/right stimulation, leading to a scale of ‘0–10’ on each side). In the choice phase, participants made trial-by-trial choices to save or spend the reward (left–right randomized cue positions). In the reward phase (following a spend choice), saved liquid rewards were delivered via computer-controlled pumps. The task allowed participants to plan their behavior up to more than 2.5 min in advance (up to 10 consecutive save choices with ~13 s cycle time, following the ~13 s planning phase). **C**, Influences on WTS ratings: reward type, interest rate and their interaction increased WTS ratings (multiple linear regression; *N* = 22; reward, *t*(21) = 4.49, *P* = 2.0 × 10^−4^; interest, *t*(21) = 5.91, *P* = 7.0 × 10^−6^; *t*(21) = 2.9, *P* = 0.009; average *R*^2^ = 0.70; average *F* = 61.2). **D**, Relationship of WTS ratings to sequence length, sequence value (inferred from choices) and (WTP, estimated by BDM auction-like mechanism). WTS correlated significantly only with sequence length and WTP, but not sequence value (random effects analysis; sequence length, *t*(21) = 15.1, *P* = 1.0 × 10^−12^; sequence value *t*(21) = −0.1, *P* = 0.9; WTP *t*(21) = 2.26, *P* = 4.0 × 10^−2^; average *R*^2^ = 0.82; average *F* = 107.6). Thus, WTP explained a component of reported intentions measured by WTS, but sequence value did not. **E**, Saving behavior in a representative participant. Bars show relative frequencies with which the participant produced different choice sequences (in other words, choices to spend at a particular sequence length). Green curves show reward magnitude increases over sequential save choices. In each plot, conditions are as follows (from left to right): low fat, low interest; high fat, low interest; low fat, high interest; high fat, high interest. The figure illustrates variations in saving behavior both across and within experimental conditions (for all plots ^*^P < 0.05, ^*^^*^P < 0.005).

## Results

The current data derive from a re-analysis of data that contributed to a previous report (Zangemeister *et al.,* 2016). The participants and behavioral methods are identical to those reported there, but we are now focusing on the assessment of intentions using a rating scale (WTS), and on the assessment of the subjective value of each sequence using a willingness-to-pay (WTP) mechanism (BDM). We have adapted the fMRI analysis to the current research questions. For reasons of completeness, we provide a full, although slightly compacted, description of all methods.

### Behavior: reported intentions (WTS)

The WTS assessed before each saving sequence was taken as indicator for saving intentions. The WTS ratings were affected by the main objective task variables reward type, interest rate and their interaction (Figure 1C; random effects multiple linear regression (‘MLR’ 1, see Supplementary Methods); *t*(21) = 4.49, *P* = 2.0 × 10^−4^; *t*(21) = 5.91, *P* = 7.0 × 10^−6^; *t*(21) = 2.90, *P* = 9.0 × 10^−3^, *t*-test; average *R*^2^ = 0.70; average *F* = 61.2). WTS ratings were greater for high-fat reward and high-interest rate compared to their lower versions. Our previous report had shown that the same two variables influenced sequence length (Zangemeister *et al.,* 2016); in a direct test, the current study found that WTS correlated with sequence length (Figure 1D). Thus, WTS appeared to be a good indicator for intentional processes related to the saving sequences of the task.

When testing the relationships to subjective value, multiple linear regression analysis revealed that WTS was significantly related to the WTP estimated by the BDM mechanism; whereas, there was no significant relationship with the sequence value inferred from observable choices included in the same regression (MLR 3; Figure 1D). Additional regression analyses showed that WTS did not predict sequence value (multiple linear regression (MLR 4); *N* = 22; sequence length, *t*(21) = 0.50, *P* = 0.62; WTS, *t*(21) = 1.1, *P* = 0.29; WTP, *t*(21) = 3.2, *P* = 4.0 × 10^−3^; average *R*^2^ = 0.67; average *F* = 37.9) and WTP was not predicted by sequence length but sequence value (multiple linear regression (MLR 5); *N* = 22; sequence length, *t*(21) = 1.43, *P* = 0.17; WTS, *t*(21) = 2.17, *P* = 0.04; sequence value, *t*(21) = 3.1, *P* = 6.0 × 10^−3^; average *R*^2^ = 0.63; average *F* = 65.1).

The relationship between WTS and WTP aligned with the similarity in the reporting nature of these two estimation procedures. For these reasons, we considered WTP as the main value regressor for the analysis of regional brain activations in the present study to investigate similarities between reported intentions and reported valuations. The WTP itself was influenced by objective variables reward type, magnitude and their interaction; WTP was greater for high-fat reward and high-interest rate compared to their lower versions (random effects multiple linear regression (MLR 2); reward type t(21) = 3.12, *P* = 5.0 × 10^−3^; magnitude *t*(21) = 7.69, *P* = 2.0 × 10^−7^; interaction *t*(21) = 3.45, *P* = 0.002; average *R*^2^ = 0.65; average *F* = 32.2). A direct comparison confirmed that WTP was greater for high- versus low-fat reward (*t*-test, *P* < 0.0001, *t*(42) = 3.72).

Taken together the behavioral analysis of WTS ratings shows that these reported intentions were best explained by an intentional component related to planned sequence length and a value component as reported in the separate task, which likely provided a basis for the reported intentions.

The saving sequences showed systematic changes with the different reward types and interest rates (Figure 1E). Higher fat content and higher interest rate were both associated with longer sequences. These relationships seemed intuitively plausible and suggested that the participants made meaningful choices during the performance of the saving sequences (Zangemeister *et al.,* 2016).

Taken together, WTS appeared to provide valid reporting for the intention to perform meaningful, self-defined saving sequences whose subjective value was related to WTP.

### fMRI: reported intentions (WTS)

**Fig. 2:**
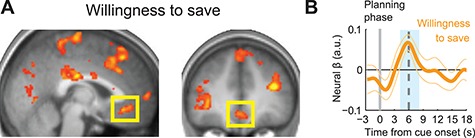
Activity in vmPFC during planning phase coding reported intentions expressed as WTS. **A**, Activity in vmPFC correlated with WTS ratings during the planning phase (*n* = 24; [−2, 34, −10], *z* = 3.68; *P* < 0.05, small volume correction on predefined coordinates). **B**, ROI analysis. Activity in vmPFC during the planning phase was related to WTS (random effects analysis; *t*(21) = 3.49, *P* = 0.002; data for this ROI analysis and all other ROI analyses were extracted from coordinates defined by leave-one-subject-out cross-validation). Neural β indicates mean regression weight from fitting a linear regression model with WTS as regressor to neural activity in each subject. Thin colored lines indicate SEM across subjects. ‘Planning phase’ indicates onset of planning phase (at 0 s). The blue shaded box indicates the analysis period at the expected delay of the hemodynamic response.

Previous research implicated the vmPFC in coding intentions and episodic prospection (Schacter *et al.,* 2007). In the current task, WTS ratings provided an explicit measure of participants’ intentions for their self-defined behavior in the forthcoming saving sequence. In a whole-brain analysis, we regressed these ratings on neural activity during the planning phase. We found that vmPFC activity during the planning phase reflected this measure of participants’ intentions (WTS; Figure 2A; Table 1, GLM1), among other brain areas including the dorsolateral prefrontal cortex (dlPFC; Table 1, GLM1). Thus, activity in both vmPFC and dlPFC encoded the participants’ reported intentions during the planning phase. Region of interest (ROI) analyses in vmPFC revealed an across-participants time course of the statistical relationship with WTS peaking around 6 s after cue onset (Figure 2B).

**Fig. 3:**
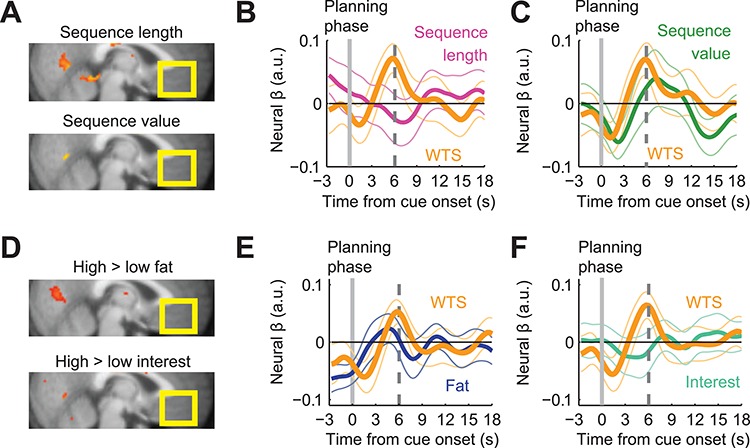
Control analyses of vmPFC activity during planning phase. **A**, Activity in vmPFC did not significantly correlate with either sequence length or sequence value. **B**, ROI analysis. Planning phase activity in vmPFC was related to WTS (random effects analysis; *t*(21) = 3.05, *P* = 0.006) but not sequence length (*t*(21) = −0.63, *P* > 0.05) during planning. **C**, ROI analysis. Planning phase activity in vmPFC was related to WTS (random effects analysis; *t*(21) = 2.61, *P* = 0.02) but not to sequence value (*t*(21) = 0.54, *P* > 0.05) during planning. **D**, Contrasts of high greater than low fat (reward type) and high greater than low interest revealed no effect in vmPFC during planning (maps thresholded at *P* < 0.005, uncorrected, voxel cluster threshold of 10 voxels). **E**, ROI analysis. Including indicator variables for fat (reward type) in a GLM with WTS during the planning phase, showed that WTS coding was unaffected and vmPFC activity did not encode reward type (WTS, *t*(21) = 2.59, *P* < 0.05; fat, *t*(21) = 0.18). **F**, ROI analysis. The equivalent analysis shown in E but for interest rate showed that WTS coding was unaffected and vmPFC activity did not encode interest (WTS, *t*(21) = 2.77, *P* < 0.05; interest, *t*(21) = −0.46).

The apparent relationship of vmPFC activity to reported intentions required a number of control tests to rule out simpler potential explanations. In a previous study, we described planning activities related to the observed sequence length and the sequence value derived from observed choices (Zangemeister *et al.,* 2016). However, the current vmPFC activity did not reflect either of these variables (Figure 3A, GLM 2 and 3, respectively). Our ROI analyses showed that the relationship with WTS was not affected by including either sequence length (Figure 3B) or sequence value (Figure 3C) as additional regressor in separate GLMs. Thus, the currently observed vmPFC activity reflected participants’ reported intentions but not the internal variables sequence length and sequence value derived from choices.

**Table 1:** Whole-brain analysis results related to parametric variables during planning phase (cluster *P*-values corrected for family-wise error across the whole brain, *P* < 0.05; maps thresholded at *P* < 0.001, extent threshold ≥10 voxels). ^*^P < 0.05, small volume corrected

Comparison	Correlation	Anatomical region	Hemisphere	Peak coordinates (mm) (*x*, *y*, *z*)	*z*-score
WTS during planning phase	Positive	Ventromedial prefrontal cortex^*^	/	-2, 34, −10	3.68
		Dorsolateral prefrontal cortex	R	38, 36, 20	4.35
		Insula	L	-30, 20, −4	4.88
		Striate cortex	R	28, −86, 2	4.65
		Visual areas 1 and 2	R	28, −78, −6	4.42
		Anterior cingulate cortex	/	2, 20, 46	4.37
		Superior temporal gyrus	R	62, −16, −10	4.37
		Posterior cingulate cortex	/	2, −18, 28	4.16
		Intraparietal sulcus	L	−40, −48, 62	3.73
WTP during planning phase	Positive	Ventromedial prefrontal cortex^*^	/	−2, 40, −8	3.54

As reported intentions were influenced by reward type (fat content) and interest rate, we tested whether vmPFC activity was directly related to either of these objective task factors. However, whole-brain control analyses revealed no significant effects in vmPFC (Figure 3D, GLM5; vmPFC significance assessed with small volume corrections). Furthermore, ROI control analyses demonstrated that inclusion of indicator functions for reward type or interest rate in separate GLMs did not affect the relationship with reported intentions (WTS; Figure 3E and F). Thus, in addition to the negative controls for sequence length and sequence value, the currently observed relationship of vmPFC activations to intention is unlikely explained by fat content and interest rate.

### fMRI results: reported value (WTP)

**Fig. 4:**
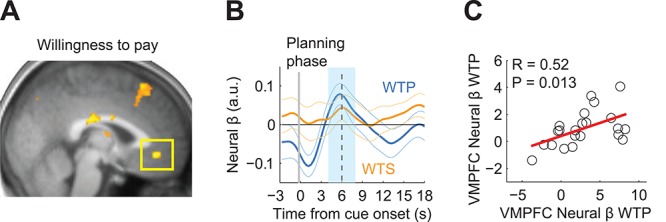
Valuation activity in vmPFC during planning phase. **A**, Activity correlated with WTP (*n* = 22; [−2, 40, −8]; *z* = 3.54; small volume correction *P* < 0.05). **B**, ROI analysis. Activity correlated with WTP (random effects analysis; *t*(21) = 2.88, *P* = 0.0089) but not WTS (*t*(21) = 1.36, *P* = 0.188). **C**, Slopes (betas) of regression of vmPFC activity on WTP and WTS across participants.

Several neuroimaging studies showed that vmPFC activities scale with the subjective value of available options at the time of choice in a variety of decision tasks (Bartra *et al.,* 2013; Clithero and Rangel, 2014), including intertemporal choice (Kable and Glimcher, 2007). In auction-like tasks, activity in vmPFC and adjoined medial orbitofrontal cortex (mOFC) scaled with participants’ liking ratings or BDM-assessed WTP of food items (Plassmann *et al.,* 2007; Plassmann *et al.,* 2010). Given these demonstrations of frontal reward value coding in very similar reporting procedures as used presently, we tested whether neural activity during the planning phase of our saving sequences scaled with participants’ WTP. Crucially, although WTS and WTP were related, each parameter explained separate parts of the variance in saving behavior (see Behavioral results). In a whole-brain GLM, we regressed WTP bids from the BDM task (Figure 4A, GLM4) on neural activity during the planning phase of the saving task (Figure 1A). This analysis revealed a significant and selective relationship between vmPFC activity and WTP for the chosen saving sequence (Figure 4B). Interestingly, unlike the WTS activations in vmPFC, dlPFC and other brain areas (Table 1), WTP activation was confined to vmPFC. Across individual participants, the peak coordinates for this relationship between WTP and vmPFC activity in the anterior–posterior axis ([−2.09 40.09 -8]) were significantly further anterior compared to those found for the relationship between WTS and vmPFC activity ([−2.09 33.73 -10], t(21) = 37.80, *P* = 8.4 × 10 − ^21^). A masking analysis confirms that the activity peak exclusively associated with WTS was located posterior to the inclusive activation (peak for WTS exclusive mask at [−2 34 -10]; inclusive mask WTS and WTP at [0 42 -8]). Taken together these results suggested that distinct activation peaks in vmPFC reflected reported valuations (WTP) and reported intentions (WTS). Across participants, slopes (betas) of regression of vmPFC activity on WTP and WTS were correlated, indicating a common coding scheme for these two introspective measures (r = 0.52, *P* < 0.05). Overall, these analyses showed that while vmPFC coded reported intentions along with other regions, it was the only structure we observed to also encode WTP.

### Activities during choice phase

**Fig. 5:**
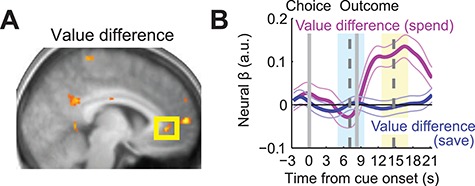
Activity in vmPFC during the choice phase. **A**, Activity in vmPFC during the choice phase was related to subjective value difference of save-spend choice options (*n* = 24; [−6, 40, −2]; *z* = 3.31; uncorrected at *P* < 0.005). **B**, ROI analysis. The vmPFC choice phase activity correlated positively with value difference during the outcome phase of spend trials (random effects analysis; *t*(21) = 5.21, *P* < 0.001).

Previous studies demonstrated vmPFC activity during choices that reflected the difference of subjective values for available options (Boorman *et al.,* 2009; Rushworth *et al.,* 2011; Kolling *et al.,* 2012; Clithero and Rangel, 2014). In the choice phase of our task, subjects were required to make trial-by-trial choices between saving the accumulated reward or spending it for immediate consumption. We previously showed that subjective values for these save and spend choice options, derived from independent behavioral data, provided a good model of participants’ choices (Zangemeister *et al.,* 2016). Accordingly, we tested in a whole-brain analysis whether this value difference was explicitly encoded by VMPFC activity in the choice phase of the saving task. We found evidence for a negative correlation with this valuation parameter in vmPFC during choices (Figure 5A, Table 2). More detailed inspection via an ROI analysis revealed that the correlation reversed from save to spend trials, becoming positive during the outcome period of spend trials (Figure 5B).

**Table 2:** Whole-brain analysis results related to parametric variables during choice phase (cluster *P*-values corrected for family-wise error across the whole brain, *P* < 0.05; maps thresholded at *P* < 0.001, extent threshold ≥10 voxels). ^*^^*^Uncorrected at *P* < 0.005

Comparison	Correlation	Anatomical region	Hemisphere	Peak Coordinates (mm) (*x*, *y*, *z*)	*z*-score
Absolute difference (sequence value—save value) during choice phase	Negative	Ventromedial prefrontal cortex^*^^*^	/	−6, 40, 0	3.46
	Negative	Posterior insula	Left	−34, −6, 8	4.48
	Negative	Superior temporal sulcus	Right	60, 2, 6	3.89

## Discussion

This study shows that activity in vmPFC in advance of a saving sequence encoded the participants’ reported behavioral intentions and the underlying, reported subjective valuation of a choice sequence. Behavioral data showed that the reported intention (WTS) correlated with the stated valuation (WTP, assessed by BDM in a separate task) of each sequence, separately from planned sequence length. The vmPFC activity correlated with the saving intention associated with each specific saving sequence, reported as WTS. Separately, vmPFC activity reflected the subjective value of each saving sequence estimated as WTP. The vmPFC intention coding was not explained by alternative task-relevant variables such as sequence length, reward type and interest rate. Thus, activity in vmPFC encoded the intention of performing a saving sequence together with the underlying valuation in advance of the sequences. Furthermore, neural betas in vmPFC for WTP and WTS were positively associated across subjects. This is intriguing as it suggests that the coding of reported intentions and valuations followed a similar scheme within different sub-regions of vmPFC.

In contrast to the current study, our previous report describes prefrontal planning activity that is related to the length of the executed sequence but occurs before the sequence starts (Zangemeister *et al.,* 2016). This activity was found in the amygdala and the dlPFC adjacent to the currently activated vmPFC. However, the previous study reported also intentional activity in the dlPFC similar to the one found presently in vmPFC. These data together suggest that substantial parts of prefrontal cortex are involved in intentional processes related to choice sequences for reward saving.

It is important to emphasize that the described planning activity occurred before commencement of the saving sequence. The separation between planning and choice phases is one of the key features of the experimental design. Stimuli during the planning phase were intentionally limited to presentation of the conditions for the upcoming saving sequence. This was done to make sure subjects were given the opportunity to plan their behavior in the absence of a choice situation or input prompt. During the choice phase on the other hand, subjects were prompted to make binary choices between saving and spending. The importance of the distinction arises, therefore, from the ability to exclusively link activity in this phase with planning without confounding events occurring simultaneously.

The results concerning subjective value reported here focus on participants’ WTP as reported in a separate task, as opposed to previously reported results that focused on subjective value inferred from choices (Zangemeister *et al.,* 2016). Interestingly, reported valuations (WTP) aligned with observed valuations (sequence value), suggesting that distinct mechanism may underlie each. We find that only WTP, not sequence value, correlated with activity in vmPFC during planning. This indicates that in the current task, vmPFC activity was associated with reported value rather than value revealed through choices. The value signals in vmPFC described here, thus, differ from sequence value signals found in the amygdala (Zangemeister *et al.,* 2016), which showed that amygdala encodes subjective value inferred from observed behavior.

The current experiment correlated neural activity in the saving task with the subjective valuation in the BDM task. Similar approaches have been successfully used in other neuroscientific studies of reward-value-guided behavior (Chib *et al.,* 2009; Hare *et al.,* 2009; Lim *et al.,* 2011). In two previous fMRI studies, participants rated reward items in one task before performing binary choices between these items in an fMRI scanner (Chib *et al.,* 2009; Lim *et al.,* 2011). For our paradigm, the use of primary rewards was essential, as they elicit ‘visceral temptations’ (Brown *et al.,* 2009) that promote variation in saving behavior and induce fMRI activations in known human reward structures (Grabenhorst *et al.,* 2010a; Grabenhorst and Rolls, 2014). However, to allow stronger conclusions on real-life valuation for saving and perhaps more complex investment options, other rewards and resources should be employed. Real-life savings and individual’s financial data could help to establish links between neural activity and financial behaviors such as thriftiness or proneness to taking on excessive debt (Benhabib and Bisin, 2005). Thus, future studies employing dynamic saving paradigms such as the one presented here might be helpful to address more realistic human-saving behavior.

The current results bear threefold significance for the common currency hypothesis, which states that vmPFC could serve as the common valuation region for choices related to many different types of reward (Hare *et al.,* 2009; Lim *et al.,* 2011; Wang and Delgado, 2019). First, observed signals were related to valuations for potential future food rewards. Second, during valuation in a BDM subjects knew that achievement of the rewards was not only delayed but also required performance of multiple saving steps to be achieved. Third, the valuations were elicited in a BDM task performed separately to the fMRI task.

Our findings also bear significance for the study of neural correlates of WTP as elicited by the BDM auction-like mechanism. Previous studies have focused on value signals during free bidding in BDM tasks. They identified activations in medial OFC and dlPFC as correlates for participants’ stated WTP for single food items (Plassmann *et al.,* 2007; McNamee *et al.,* 2013). Our results lend further support for the validity of using this WTP method to elicit neural value signals. Furthermore, the systematic and meaningful bids for combinations of saving steps, reward type and interest rate suggest that the BDM mechanism is valid also for rewards with multiple and dissociable components resulting from multi-step choice sequences. This finding provides evidence that WTP estimates can be used as an integrative value parameter across reward dimensions, opening up further avenues for studying neural mechanisms of reward in humans.

Earlier studies suggested an involvement of vmPFC in imagining future states (‘episodic prospection’) (Schacter *et al.,* 2007) and in representing goal states during hierarchical navigation (Balaguer *et al.,* 2016). These data suggest a role for this brain structure in the pursuit of future goals. During value-based choices, vmPFC activity signals the subjective value of attended and chosen options (Boorman *et al.,* 2009; Lim *et al.,* 2011; Rushworth *et al.,* 2011; Bartra *et al.,* 2013; Clithero and Rangel, 2014), including temporally discounted values of future rewards (Kable and Glimcher, 2007). Such valuations of future rewards by vmPFC are also modulated by episodic prospection (Peters and Buchel, 2010). Signals in vmPFC influence planning through interactions with striatum during multi-step reinforcement learning (Wunderlich *et al.,* 2012) and code value during dynamic persistence (McGuire and Kable, 2015). Our results confirm the general involvement of vmPFC in these intentional and valuation processes.

Our paradigm is interesting in several ways. First, the economic-saving task included three key phases: planning, decision and reward outcome. These phases were separated temporally, allowing for analysis and comparison of task-related activities using multiple linear regressions with well-distinct regressors. The analyses afforded by the well-separated task phases identified distinct vmPFC signals for intention and valuation during the planning phase, as opposed to other task phases of less interest. Second, valuations were assessed using an incentive compatible mechanism that encouraged truthful subjective estimates. Third, as valuations, intentions were assessed using a similarly explicit report that involved subjective ratings on a continuous scale. These similarities in the eliciting procedures may explain the interesting finding of valuation and intention mechanisms in the same prefrontal area.

## Supplementary Material

scan-19-120-File002_nsaa013
