# Supplementary material for: Neural activity in human ventromedial prefrontal cortex reflecting the intention to save reward
Source: Soc Cogn Affect Neurosci. 2020 Jan 28;14(12):1255–61. doi: 10.1093/scan/nsaa013 (PMC7137725; doi:10.1093/scan/nsaa013)
Supplement: scan-19-120-File002_nsaa013 [file scan-19-120-file002_nsaa013.docx]

## **SUPPLEMENTARY MATERIAL**

## **Material and Methods**

**Participants**

Twenty-eight healthy individuals (18-33 years; 13 females) with normal or corrected-to-normal vision participated in the study. We excluded four participants from all analyses due to motion artefacts, giving a sample size of n = 24, and we excluded two further participants from the BDM task due to incomplete data sampling, resulting in 22 participants in whom we performed our complete set of analyses.Participants tolerated lactose and dairy products, did not avoid fat and sugar diests, had normal appetite, were not pregnant, had no psychiatric history, no recent medication except contraceptives, were healthy according to self-report, and gave their written consent before the experiment. The Local Research Ethics Committee of the Cambridgeshire Health Authority approved the study.

**Experimental design**

Before scanning, each participant took part in a behavioral session on a separate occasion. During this session, participants performed exactly the same task as in the scanner, including delivery of the liquid rewards. Participants were asked to not eat or drink anything except water for at least 4 hours before each session. This was done to ensure that participants were hungry and willing to perform a task towards gaining liquid food rewards. Stimuli were presented on a computer monitor, and responses were given by pressing specific keys on a keyboard (pre-scan session) or button box (scanning session). Stimulus presentation and operant reactions were controlled and recorded using Cogent (Wellcome Trust Centre for Neuroimaging, London, UK) in Matlab (Version R2013b, Mathworks, Natick, MA).

**Economic saving task**

Participants performed choice sequences of self-defined lengths to save different liquid rewards. Each sequence comprised three phases, consisting of the planning phase during which we analysed BOLD signals in relation to the intention to save (willingness-to-save, WTS) and the subjective value of the sequence (willingness-to-pay, WTP), followed by the chosen series of save-spend choices, and the final reward phase (Fig. 1A). In a 2×2 factorial design, we used two different reward types (high vs. low fat content) and two interest rates (high vs. low) with which the rewards accumulated over successive save trials (Fig. 1B). Note that we use the term ‘interest rate’ to provide an intuitive description of the variable that governed increases in reward across save choices; this should not imply exact comparability with financial interest rates. We used this design with the aim to promote variation in participants’ saving behavior and to distinguish neural activity related to sequence length (a linear function of the number of sequential save choices) from activity related to sequence value (which depended on participants’ preferences for different reward types, reward amounts and their interaction). All frame durations in the task were jittered according to Poisson distributions with an additional jitter of ± 200 ms to avoid predictability and to increase fMRI image acquisition efficiency. The experiment was divided in three consecutive sessions of 20 minutes each with a brief break in between (total time therefore 60 minutes). On average, participants performed 42.6 ± 1.4 saving sequences (mean ± S.E.M.) during the fMRI experiment, with an average of 213 ± 2.8 save-spend choice trials. These numbers exclude error sequences and error trials (see below), which were also excluded from all fMRI analyses. *Planning phase.* During the planning phase (Fig. 1A), pre-trained cues indicated current interest rate (high vs. low) and current reward type (high vs. low fat content). The order of conditions (combinations of reward type and interest rate) between sequences was pseudo-randomized to avoid predictability but ensuring even numbers of each condition. With each sequence, an initial cue indicated the reward type and interest rate. The cue lasted for 2-3 s; at 2-4 s following cue offset, a visual analogue scale ranging from 0 (low WTS) to 10 (high WTS) appeared, titled ‘Willingness to save’, upon which participants rated within 3.0 s their WTS (Fig. 1A, left). The participants had received a written instruction to indicate ‘how willing are you to save?’. The WTS stated by the participants before each sequence correlated with the length of that performed sequence (R^2^ = 0.58±0.13; Kendall’s Tau rank correlation). Despite this level of correlation, there was sufficient variation between WTS and sequence length to allow for detection of distinct neural correlations. The WTS rating was followed by the start of the choice phase after an interval of 2-4 s.

*Choice phase.* During the choice phase (Fig. 1A), participants made trial-by-trial choices to save more reward or spend the accumulated reward. Each choice trial began with the presentation of a question mark on the screen for 2-3 s which prompted participants to consider their save vs. spend choice for that trial. Following an interval of 2-4 s, the save and the spend cues appeared in left-right positions; participants indicated their choice with a button press at a position corresponding to the chosen cue. The left-right position of save and spend cues was randomized across trials. Button presses were self-timed but required a choice within 3 s. A save choice was followed by a 2-3 s feedback screen stating ‘Saved’, without providing feedback about saved reward amount. Thus, the participants had to track internally the accumulated reward amounts over consecutive save choices. Consecutive choice trials were separated by inter-trial intervals of 2-6 s. In each saving sequence, participants were required to make at least one save choice before making a spend choice to receive a reward. The maximal number of consecutive save choices per sequence was 10; the cycle time per trial was approximately 13 s (trial duration plus inter-trial interval). A failure to respond on any trial led to an error feedback stating ‘Please repeat trial’ and resulted in the repetition of the trial. Accumulated saved rewards were retained across error trials. If a participant made more than the allowed ten save choices in a sequence, they received the feedback ‘Saved too long’, which resulted in cancellation of the saving sequence. This error occurred only rarely during the scanning experiment (mean = 1.08 ± 0.2) as participants were pre-trained.

*Reward phase.* The reward phase followed participants’ spend choice in each sequence (Fig. 1A). A spend choice was followed by a 2-3 s feedback screen stating ‘Receive X ml in 2 sec’. The accumulated amount of liquid reward was then delivered via a custom-made system consisting of two peristaltic pumps (see below). After reward delivery participants were instructed to keep the liquid in their mouth for 0.5 s before swallowing for 1.5 s. Reward delivery and swallowing periods were cued by a yellow and green fixation cross, respectively. Participants then rated the experienced pleasantness of the liquid on a visual analogue scale ranging from of 0 (very unpleasant) to 10 (very pleasant). The general protocol and procedures for liquid reward delivery in the scanner were modelled on previous fMRI studies ([Grabenhorst et al., 2010a](#_ENREF_14); [Grabenhorst and Rolls, 2014](#_ENREF_17)).

*Liquid rewards.* The liquid rewards consisted of lightly cooled vanilla-flavoured dairy drinks that differed in fat content. The low fat version contained 400 ml of skimmed milk (0.2 % fat), whereas the high fat version consisted of 300 ml double cream and 100 ml full fat milk (total of 34.5% fat). Total sugar content was equal for both drinks. 10 ml of vanilla extract was added to each drink. The stimuli resembled those used in previous human fMRI studies in which they potently activated major reward areas ([Grabenhorst and Rolls, 2014](#_ENREF_17); [Grabenhorst et al., 2010b](#_ENREF_18)).

*Reward delivery.* The accumulated amount of liquid reward was delivered via peristaltic pumps (Experimental Psychology Workshop, University of Cambridge) placed outside the scanner room. They were connected to a computer using an external National Instruments card (NI-USB-6009, National Instruments, Austin, Texas) and controlled via custom-made software using the Matlab Data Acquisition Toolbox. Participants received the liquid through a custom-made mouthpiece that was connected to silicone tubes of about 10 m length explicitly suitable for foodstuff (VWR International Ltd, UK).

**Interest rate calculation**

Growth in reward over consecutive save choices was calculated according to

$$x_{n}= b\sum_{i=0}^{n-1} q^{i}$$

with $x_{n}$ as reward magnitude on trial$n$, $b$ as the base rate of reward magnitude, and $q$ as the interest rate ([Grabenhorst et al., 2012](#_ENREF_15); [Hernadi et al., 2015](#_ENREF_20)). The interest rate was either high ($q=1.3$) or low ($q=0.9$), resulting in a quasi-hyperbolic growth profile for the low $q$ and quasi-exponential growth profile for the high$q$ (green curves in Fig. 4.1D). Base rate was set to $b=0.11$. Interest rates and reward magnitudes were chosen based on behavioral pre-testing to ensure that participants could discriminate the different reward magnitudes and were still able to drink the highest reward magnitude (6.2 ml) in the scanner. The following provides an example of how reward magnitudes were calculated. With a base rate of *b* = 0.11 and an interest rate of *q* = 1.3, on the first trial of the choice sequence the reward magnitude (RM) would correspond to RM = 0.11 × (1 + 1.3) = 0.25 ml. On the second trial, with two successive save choices, RM = 0.11 × (1 + 1.3 + 1.3^2^) = 0.44 ml. On the third trial, with three successive save choices, RM = 0.11 × (1 + 1.3 + 1.3^2^ + 1.3^3^) = 0.68 ml. The interest rate calculation adopted for this experiment does not exactly match calculations commonly employed in financial theory. The definition described above was used in order to yield a decreasing marginal increase of reward for the low interest condition.

**Valuation of sequences**

The behavioral task comprised 40 different sequences, which consisted of 10 possible sequence lengths defined by the maximal number of consecutive save choices, 2 reward types (low/high fat) and 2 interest rates (low/high). The value of each of the 40 sequences depended on the final reward amount that was obtained from performing the sequence with chosen length, reward type and interest rate. In addition to this objective measure of value, each sequence length was associated with a different amount of temporal discounting and a different physical effort. Rather than dissecting the complex subjective influences of these measures on the participants' behavior and brain activity, which would have exceeded our present aims, we estimated the subjective value of each of the 40 sequences, using two specific behavioral procedures.

*Sequence value by willingness-to-pay ('WTP').* On different days and outside of the scanner, we estimated the subjective value separately for each sequence as WTP by implementing a Becker-DeGroot-Marschak auction-like mechanism (BDM) ([Becker et al., 1964](#_ENREF_4)). This procedure resembled the reporting nature of the WTS estimation of the participant's intention to save; in support of this similarity, WTS correlated significantly with WTP (Fig. 1D). An example sequence for WTP estimation would be ‘save 7 times to receive 2.6 ml of the high fat reward’. Information about the required number of save choices and the available reward amounts were provided in text form, whereas information about reward type was shown in the same way as in the main saving task, i.e. using a colored cue. After viewing the current option, the word ‘bid?’ appeared below the option, followed by a response by the participant in the range of 1 (low) to 5 (high) on a keyboard. Participants were informed that a small number of auctions would be randomly selected by the computer and payed out after the experiment. Three such auctions were randomly selected among trials 5-10, 15-20 and 25-30, respectively.

To run each BDM estimation, the participant placed a bid. This bid was compared to a randomly generated number between 1 and 5 that served as computer bid. If the participant’s bid was higher or equal to the computer bid, the participant ‘won’ the auction. Winning the auction resulted in guided performance of the choice sequence that the participant had bid for. Each participant received a specific number of points as endowment; the remainder of the endowment was converted into drink after the task at an exchange rate of 1 point to 0.5 ml of drink (low fat reward type, skimmed milk). Volunteers were carefully instructed about the rules of the task. Post-instruction questionnaires confirmed that participants understood the task rules and the different choice options; they found the description of the task intuitive in terms of save-spend decisions.

*Sequence value by observable choice frequency ('sequence value').* This estimation procedure differed from the WTP and WTS assessments in deriving subjective sequence value from behavioral choices in each sequence during scanning, as used before ([Grabenhorst et al., 2012](#_ENREF_15); [Hernadi et al., 2015](#_ENREF_20)). The methodological difference corresponds to the lack of correlation between WTS and sequence value derived from choices (Fig. 1D). To estimate sequence value, we followed the general notion of economic choice theory according to which one infers (unobservable) value from observable choices. This approach is straightforward when every trial involves a choice between two explicit options; the value of an option is estimated as a real-number multiple of the amount of a common currency reference option ('numeraire') at choice indifference ([Lak et al., 2014](#_ENREF_23)). By contrast, for the present experiment, we aimed to obtain a subjective value estimate for a whole sequence but there was no choice between two sequences; rather, each sequence required sequential choices until the final spend option was selected. We therefore defined the subjective value from the relative frequency (from 0 to 1) with which each participant chose the spend option at each sequence step, separately for each of the two reward types and each of the two specific interest rates. The frequencies of each of these four types of sequence summed up to 1.0. We then multiplied the choice frequency at each of the 10 steps with the objective reward magnitude obtained by choosing the spend option at that step (Fig. 1E, green curves). This measure of 'sequence value' was subjective as it rested on the (subjective) choice frequencies of the animal. Thus, the sequence value gained in a given saving sequence was based on the subjective value of the saving sequence as inferred from choices. Thus, there were 40 different sequence values (10 sequences according to the number of save choices before spend choice, 2 reward types, 2 interest rates).

Formally, the subjective value for spending at any step $i$ in the choice sequence was defined as

$${Sequence value}_{i}=P_{i} \times{RM}_{i}$$

with $P_{i}$ as the relative frequency of observing a spend choice at a given point *i* in a saving sequence (defined by the number of consecutive save choices) and with $RM_{i}$ as the reward magnitude (in ml) delivered after the spend choice on that step. This definition of sequence value as relative choice frequency weighted by reward magnitude incorporated value differences between interest rates: for the high interest rate, a given sequence length was associated with more reward compared to the low interest rate, which resulted in higher subjective value (assuming a positive monotonic subjective value function on magnitude). The high fat reward was associated with longer sequences, and thus higher subjective value, compared to low fat reward (Fig. 1E yellow vs. blue).

To model trial-by-trial save-spend choices, we defined the value of a save choice at a given position in a saving sequence (‘save value’) as the average sequence value associated with all potential future trials of that sequence. Thus, the subjective value for saving at a given point *n* in a sequence was

$${Save value}_{n}= \frac{1}{m-n}\sum_{i=n+1}^{m} {Sequence value}_{i},$$

with *m* defining the upper limit of the saving sequence (given by the maximal observed sequence length for the participant and condition). Thus, ‘current sequence value’ and ‘save value’ reflected trial-by-trial valuations, whereas ‘sequence value’ constituted the value of the finally chosen sequence.

**Multiple linear regression analysis of behavior**

We calculated a model to understand the contributions of objective factors on WTS ratings (MLR 1):

$${WTS=\beta}_{0}+\beta_{1}RewardType+\beta_{2}Interest+\beta_{3}RewardType\times Interest+\varepsilon$$

with $RewardType$ as the current reward type (dummy variable for high vs. low fat content, with 1 indicating high fat and 0 indicating low fat), $Interest$ as the current interest rate (dummy variable for high vs. low interest rate with 1 indicating high interest and 0 indicating low interest),$RewardType \times Interest$ as interaction term, $\beta_{0}$as constant term, $\beta_{1}$to $\beta_{3}$as the corresponding slope parameter estimates, and $\varepsilon$ as residual. The results are shown in Fig. 1C.

To model the influences of objective factors interest rate and reward type on WTP-bids in the auction task, we performed the following multiple linear regression (MLR 2):

$${WTP=\beta}_{0}+\beta_{1}Reward Type+\beta_{2}Reward Magnitude+\beta_{3}Reward Magnitude\times Reward Type+\varepsilon$$

with $Reward$ as the current reward type (dummy variable for high vs. low fat content, with 1 indicating high fat and 0 indicating low fat), $Reward Magnitude$ as the magnitude in ml of the saving strategy option, $Reward Magnitude\times Reward Type$ as interaction term, $\beta_{0}$as constant term, $\beta_{1}$to $\beta_{3}$as the corresponding slope parameter estimates, and $\varepsilon$ as residual.

To assess the relationships between WTS ratings, sequence length, sequence value and WTP we performed the following multiple linear regression analyses (MLR 3).

$${WTS=\beta}_{0}+\beta_{1}Sequence length+\beta_{2}Sequence value+\beta_{3}WTP+\varepsilon$$

And (MLR 4)

$${Sequence value=\beta}_{0}+\beta_{1}Sequence length+\beta_{2}WTS +\beta_{3}WTP+\varepsilon$$

And (MLR 5)

$${WTP=\beta}_{0}+\beta_{1}Sequence length+\beta_{2}WTS +\beta_{3}Sequence value+\varepsilon$$

With $Sequence length$ as the observed length of the forthcoming sequence, $Sequence value$ as the subjective value for the forthcoming sequence, $WTP$as the reported valuation elicited in a separate task, $\beta_{0}$as constant term, $\beta_{1}$to $\beta_{3}$as the corresponding slope parameter estimates, and $\varepsilon$ as residual.

**fMRI data acquisition**

We acquired echo T2*-weighted echo-planar images (EPIs) with blood-oxygen-level-dependent (BOLD) contrast using a Siemens 3T Trio Scanner at the Wolfson Brain Imaging Centre, Cambridge, UK. Data were acquired with in plane resolution 3 × 3 × 2 mm, 2 mm slice thickness, 56 slices, repetition time (TR) = 3 s, echo time (TE) = 30 ms, flip angle = 90° and field of view = 192 mm. Between 401 and 470 volumes were acquired in three separate runs for each participant, along with 4 ‘dummy’ volumes before each scanning run. The acquisition plane was tilted by -30 degrees with respect to the anterior commissure–posterior-commissure axis and a z-shim gradient pre-pulse was applied to minimize signal dropout in inferior frontal and medial temporal lobe areas ([Deichmann et al., 2003](#_ENREF_11)). High-resolution T1 structural scans were acquired using an MPRAGE sequence and co-registered to enable group level anatomical localization with the following sequence parameters: 1 × 1 × 1 mm^3^ voxel resolution, 1 mm slice thickness, TR = 2.3 s, TE = 2.98 ms, inversion time 900 ms, flip angle = 9°.

**fMRI Data analysis**

We performed the fMRI data analysis using statistical parametric mapping (SPM8; Wellcome Trust Centre for Neuroimaging, London). Pre-processing included realignment of functional data including motion correction, normalization to the Montreal Neurological Institute (MNI) coordinate system, and smoothing with a Gaussian kernel with full width at half maximum (FWHM) of 6 mm. A high-pass temporal filter with a cut-off period of 128 s was applied. General linear models (GLMs) assuming first-order autoregression were applied to the time course of activation in which event onsets were modelled as single impulse response functions convolved with the canonical hemodynamic response function. Time derivatives were included in the basis functions set. Linear contrasts of parameter estimates were defined to test specific effects in each individual dataset. Voxel values for each contrast resulted in a statistical parametric map of the corresponding *t* statistic. In the second (group random-effects) stage, subject-specific linear contrasts of these parameter estimates were entered into one-sample t-tests and tested positive (e.g. corresponding to positive relationship between WTS and neural activity) or negative contrasts, as described below, resulting in group-level statistical parametric maps.

We estimated the following GLMs to test specific hypotheses:

*GLM 1.* This GLM served two purposes: (1) to search for regions correlating with the WTS ratings during planning. For each participant we estimated a GLM with the following regressors of interest: (R1) an indicator function for the choice phase, i.e. the times when participants were presented with the question mark cue prompting them to consider their save-spend decision for the current trial; (R2) R1 modulated by the willingness to save indicated before the sequence; (R3) an indicator function for the action phase, i.e. the times when the save and spend cue were presented and the participant could enter their choice using the button box; (R4) R3 modulated by an indicator function indicating whether the participant chose the cue presented on the left or on the right; (R5) an indicator function for the planning phase, i.e. the times when cues indicating interest rate and reward type were shown; (R6) R5 modulated by the WTS ratings for the forthcoming sequence; (R7) an indicator function for the WTS rating phase, i.e. the times when participants indicated their WTS on a visual analogue scale from 0 (low) to 10 (high); (R8) an indicator function for the reward delivery period, i.e. the times when reward was delivered into the participant’s mouth; (R9) R8 modulated by the reward magnitude (in ml); (R10) an indicator function for the pleasantness-rating phase, i.e. the times when the participants indicated the pleasantness of the received reward; (R11-R17) the motion parameters resulting from the realignment pre-processing step as covariates of no interest; (R18-R20) three session constants.

*GLM 2.* This GLM served to search for regions correlating with the final length of the current choice sequence during the choice phase. For each participant we estimated a GLM with the following regressors of interest: (R1) an indicator function for the choice phase, i.e. the times when participants were presented with the question mark cue prompting them to consider their save-spend decision for the current trial; (R2) R1 modulated by the final sequence length of the current choice sequence; (R3) an indicator function for the action phase, i.e. the times when the save and spend cue were presented and the participant could enter their choice using the button box; (R4) R3 modulated by an indicator function indicating whether the participant chose the cue presented on the left or on the right; (R5) an indicator function for the planning phase, i.e. the times when cues indicating interest rate and reward type were shown; (R6) R5 modulated by the length of the forthcoming choice sequence (‘sequence length’); the remaining details were the same as in GLM1. The full results for this GLM have been reported previously ([Zangemeister et al., 2016](#_ENREF_38)).

*GLM 3.* This GLM identified regions associated with sequence value. It included the following regressors: (R1) an indicator function for the choice phase; (R2) R1 modulated by the sequence value (i.e. the final chosen sequence value) of the current choice sequence. (R3) an indicator function for the action phase; (R4) R3 modulated by an indicator function indicating whether the participant chose the cue presented on the left or on the right; (R5) an indicator function for the planning phase; (R6) R5 modulated by sequence value; (R7) an indicator function for the WTS rating phase; (R8) an indicator function for the reward delivery period; (R9) R8 modulated by the pleasantness rating; (R10) an indicator function during the pleasantness-rating phase. The remaining details were the same as in GLM1. The full results for this GLM have been reported previously ([Zangemeister et al., 2016](#_ENREF_38)).

*GLM 4.* This GLM served to search for regions correlating with the willingness to pay bids for the forthcoming sequence; For each participant we estimated a GLM with the following regressors of interest: (R1) an indicator function for the choice phase, i.e. the times when participants were presented with the question mark cue prompting them to consider their save-spend decision for the current trial; (R2) R1 modulated by the willingness to pay for the current sequence; (R3) an indicator function for the action phase, i.e. the times when the save and spend cue were presented and the participant could enter their choice using the button box; (R4) R3 modulated by an indicator function indicating whether the participant chose the cue presented on the left or on the right; (R5) an indicator function for the planning phase, i.e. the times when cues indicating interest rate and reward type were shown; (R6) R5 modulated by the reward magnitude of the current sequence; The remaining details were the same as in GLM1.

*GLM 5.* This GLM served to test whether parametric effects during the planning phase could be explained by the objective factors fat and interest (Fig. 4.9A, B). The model contained the following regressors: (R1) an indicator function for the choice phase; (R2) R1 modulated by current sequence length; (R3) an indicator function for the action phase; (R4) R3 modulated by an indicator function indicating whether the participant chose the cue presented on the left or on the right; (R5) an indicator function for the planning phase in the low interest, low fat condition; (R6) R5 modulated by sequence length; (R7) an indicator function for the planning phase in the high interest, low fat condition; (R8) R7 modulated by sequence length; (R9) an indicator function for the planning phase in the low interest, high fat condition; (R10) R9 modulated by sequence length; (R11) an indicator function for the planning phase in the high interest, high fat condition; (R12) R11 modulated by sequence length. The remaining details are the same as for GLM1.

*GLM 6.* This GLM served to search for regions correlating with subjective value difference during choices; For each participant we estimated a GLM with the following regressors of interest: (R1) an indicator function for the choice phase, i.e. the times when participants were presented with the question mark cue prompting them to consider their save-spend decision for the current trial; (R2) R1 modulated by the absolute difference between sequence value and save value on the current trial; (R3) R1 modulated by the final sequence value of the current sequence; (R4) an indicator function for the action phase, i.e. the times when the save and spend cue were presented and the participant could enter their choice using the button box; (R5) R4 modulated by an indicator function indicating whether the participant chose the cue presented on the left or on the right; (R6) an indicator function for the planning phase, i.e. the times when cues indicating interest rate and reward type were shown; (R7) R6 modulated by the final sequence length of the forthcoming sequence; The remaining details were the same as in GLM1.

**Statistical significance testing and localisation**

For all fMRI analyses, we report effects that survive correction for multiple comparisons across the whole brain using a significance level of P < 0.05 (family-wise error) at cluster level. This was imposed on maps that were displayed at P < 0.001 with minimum cluster size *k* = 10 voxels. In addition, we used small volume correction (P < 0.05, cluster-level) in vmPFC. We defined these coordinates for small volume corrections a priori. Specifically, we used the coordinates [-2, 40, -6] based on a recent meta-analysis on reward valuation ([Clithero and Rangel, 2014](#_ENREF_10)).

**Region of interest analysis: BOLD time courses**

For the analysis in vmPFC, we extracted raw BOLD data around the same independent coordinates used for small volume correction [-2, 40, -6] ([Clithero and Rangel, 2014](#_ENREF_10)). Following data extraction we applied a high-pass filter with a cut off period of 128 s. The data were then z-normalized, oversampled by a factor of 10 using sinc-interpolation and separated into trials to produce a matrix of trials against time. We generated separate matrices for each event of interest (e.g. onset of planning phase). The resulting BOLD plot is shown in Fig. 2C.

**Region of interest analysis: Effect size time courses**

To calculate effect size time courses we took the BOLD data and fitted the GLMs to each oversampled time point across trials separately in each participant. The GLMs were designed to test specific hypotheses as described in the text. In addition to the regressors shown in each figure, the GLMs included motion parameters as covariates of no interest. This GLM analysis yielded one regression coefficient for each regressor for every oversampled time point in each participant. We entered individual-participant coefficients into one-sample t-tests (random-effects analysis, P < 0.05) and calculated group averages and standard errors for each time point across participants, yielding the across-participant effect size time courses shown in the figures. These mean effect size time courses are shown for vmPFC in Figs. 2B,3B-C, 3E-F 4B, 5B.

**Shared variance and relationship between our main variables**

As reported previously ([Zangemeister et al., 2016](#_ENREF_38)), we calculated the shared variance between our main regressors for fMRI data analysis within each participant. The shared variances were as follows: WTS and WTP: 0.56; WTS and Sequence length: 0.88; WTS and Sequence value: 0.55; WTP and Sequence length: 0.58; WTP and Sequence value: 0.49; Sequence length and Sequence value: 0.65; Sequence length and BDM bids: R^2^ = 0.36 (± 0.28); sequence value and BDM bids: R^2^ = 0.37 (± 0.28).

**Data and code availability**

All relevant data and the code for the analyses are available from the authors upon reasonable request.
